# Variation in colon cancer survival for patients living and receiving care in London, 2006–2013: does where you live matter?

**DOI:** 10.1136/jech-2021-217043

**Published:** 2021-08-16

**Authors:** Manuela Quaresma, James R Carpenter, Adrian Turculet, Bernard Rachet

**Affiliations:** 1 Non-Communicable Disease Epidemiology, London School of Hygiene & Tropical Medicine, London, UK; 2 Medical Statistics, London School of Hygiene & Tropical Medicine, London, UK; 3 London Hub for Trials Methodology Research, MRC Clinical Trials Unit at UCL, London, UK

**Keywords:** geography, health policy, inequalities, multilevel modelling, neoplasms

## Abstract

**Background:**

Marked geographical disparities in survival from colon cancer have been consistently described in England. Similar patterns have been observed within London, almost mimicking a microcosm of the country’s survival patterns. This evidence has suggested that the area of residence plays an important role in the survival from cancer.

**Methods:**

We analysed the survival from colon cancer of patients diagnosed in 2006–2013, in a pre-pandemic period, living in London at their diagnosis and received care in a London hospital. We examined the patterns of patient pathways between the area of residence and the hospital of care using flow maps, and we investigated whether geographical variations in survival from colon cancer are associated with the hospital of care. To estimate survival, we applied a Bayesian excess hazard model which accounts for the hierarchical structure of the data.

**Results:**

Geographical disparities in colon cancer survival disappeared once controlled for hospitals, and the disparities seemed to be augmented between hospitals. However, close examination of patient pathways revealed that the poorer survival observed in some hospitals was mostly associated with higher proportions of emergency diagnosis, while their performance was generally as expected for patients diagnosed through non-emergency routes.

**Discussion:**

This study highlights the need to better coordinate primary and secondary care sectors in some areas of London to improve timely access to specialised clinicians and diagnostic tests. This challenge remains crucially relevant after the recent successive regroupings of Clinical Commissioning Groups (which grouped struggling areas together) and the observed exacerbation of disparities during the COVID-19 pandemic.

## Introduction

Population-based cancer survival statistics provide key insights into the overall effectiveness of a healthcare system in managing and treating patients with cancer.^1^ Quantifying disparities in cancer survival in particular can directly identify areas of inequity amenable to change. For instance, wide geographical and socioeconomic inequalities in cancer survival have been consistently described, despite the existence of universal access to care within the National Health Service (NHS), founded on the principles of equity and free access to all.^2–5^ A clear and persistent North–South gradient, with lower survival in the North of England, has been observed for most common adult cancer types, with similar patterns reported within London, almost mimicking a microcosm of the country’s survival patterns. This evidence has suggested that the place of residence might play an important role in the survival of a patient with cancer, giving rise to much political debate since the introduction of the first NHS cancer plan and other national initiatives aimed at tackling cancer inequalities.^6–8^ Studying geographical variations in health outcomes is challenging as English health geographies have continuously changed over the last decades, through mergers, boundary changes, creation and cessation of geographies.^9 10^ Following the 2012 Health and Social Care Act and the subsequent restructuring of the NHS, two organisations became central role players in the organisation and commissioning of care: NHS England and Clinical Commissioning Groups (CCGs).^11^ NHS England became responsible for commissioning the planning and buying of healthcare services, such as primary care services, and setting the priorities and direction of the NHS. It also allocates 60% of the NHS budget to CCGs across England. CCGs are clinically led statutory NHS bodies responsible for the planning and commissioning of healthcare services for their local area, including general practitioner (GP) services, planned hospital, urgent and emergency care. More recently, CCGs have been merged, from the initial 212 to 135 in 2020, and further to 106 from 1 April 2021. Cancer survival outcomes for CCGs have been published on a regular basis since their creation, including an index of cancer survival for all cancers combined and cancer-specific survival indexes for breast, colorectum and lung cancers.^12^ These outcomes have provided evidence of wide geographical variation in cancer across England, as well as within London. Understanding the mechanisms underlying such wide disparities requires addressing multiple research questions to disentangle the different aspects of the multilayered and multifactorial ‘cancer inequalities puzzle’, including the integrated study of patient-system, tumour-system and health-system characteristics. In this article, we examine the geographical variations in colon cancer outcomes among patients living in London (England) in order to examine whether geographical variation in cancer survival is associated with the hospital of care. We use the catchment area of each CCG (as defined in 2013) as the geographical unit of analysis. We start by investigating the patterns of patient pathways between the area of residence and the NHS hospital of cancer care. Next, we investigate the variability in cancer survival at both CCG and hospital level after adjusting for some patient and tumour characteristics, such as age at diagnosis, socioeconomic status and stage at diagnosis.

## Material and methods

### Data

Data on individual cancer records were obtained from the National Cancer Registry at the Office for National Statistics for all adults (aged 15–99 years) diagnosed with a first, primary, invasive malignancy of the colon during 2006–2013 in London, England. All patients were followed up to update their vital status until 31 December 2014. The data variables available for analysis from this data source were gender, age at diagnosis, full dates of diagnosis, last follow-up and death, vital status indicator (dead or censored as alive at the end of follow-up), CCG of residence at diagnosis, deprivation category (1—least deprived to 5—most deprived), colon cancer stage at diagnosis (1—localised cancer stage to 4—metastatic cancer stage) and routes to diagnosis (screen-detected, 2-week wait, emergency presentation, GP referral, inpatient elective, other outpatient, death certificate only, unknown).^13 14^ A CCG of residence was allocated to each patient based on his/her postcode of residence. Deprivation categories (1—least deprived to 5—most deprived) were defined according to the quintiles of the distribution of the Income Domain scores of the 2011 Indices of Multiple Deprivation (IMD) in all Lower Super Output Areas (LSOA) in England. Each patient was then allocated to one of these five deprivation categories based on his/her LSOA of residence at the time of his/her diagnosis. We selected the Income Domain as the ecological measure of deprivation because it tries to quantify material wealth (as opposed to housing, educational or health deprivation) and is thus more comparable with measures of material deprivation. It has been shown that socioeconomic inequalities were well measured using the Income Domain of the IMD and were consistent with other commonly used measures such as Carstairs.^15^ To complement the cancer registry dataset with information on stage at diagnosis and hospital of cancer care, each individual cancer record was linked to two additional sources of data, Hospital Episode Statistics (HES) records and the National Bowel Cancer Clinical Audit data, using a data linkage algorithm by Shack *et al*.^16^ After the three data sources were linked, the stage at diagnosis variable was reconstructed using the algorithm by Benitez Majano *et al* 17 that combines available information on tumour (T), nodes (N) and metastases (M). The algorithm prioritises information captured in the clinical audit data and, if not available, uses cancer registry stage data. Treatment information was also derived from clinical audit data and HES records using an algorithm by Fowler *et al* 18 that categorises major surgical treatment received by each patient within a time window of between 30 days prior and 90 days following cancer diagnosis, and categorises other minor forms of treatment (including palliative care and diagnostic procedures if no other treatment was recorded) into a minor treatment category. Based on the previous definition of treatment categories, we allocated to each patient with cancer a hospital of cancer care, or diagnosis if no major surgical treatment was received, using a combination of different variables available in the data containing hospital codes.

### Statistical methods and data visualisation

In addition to usual descriptive statistics calculated in Stata software V.15,^19^ various data visualisation techniques were used. Windrose graphs were used to display the distribution of patients’ deprivation category and stage at diagnosis by CCG of residence and hospital of cancer care. CCGs and hospitals were arranged in the windroses according to their approximate cardinal directions of location in London for ease of visualisation. Flow maps of London were created to visualise patterns of patient pathways between the CCG of residence and the hospital of cancer care. The maps show the areas of catchment and boundaries for each of the 32 London CCGs, all identified with their names. The 36 London NHS hospitals used in this study are marked on the maps using the exact location based on their latitude and longitude coordinates. All these hospitals include tertiary referral departments. The key to the hospital names is given in the map legend using the identifiers (H1, H2, …, H36). Each pathway is shown on the map using lines connecting the centroid of each CCG (black dot) to each hospital. The pathway line colours distinguish between the frequency of each pathway, coloured from the most frequent up to the fifth most frequent, with the proportion (%) of patients using each pathway indicated on the lines. Only pathways that had more than 5% of patients were drawn, and thus, the sum of all the pathway frequencies originating from each CCG will not add to 100%. Maps were created using the software ArcGIS V.10.5.^20^

In order to investigate the variability in cancer survival at CCG and hospital levels, net survival (survival from the cancer) and excess hazard of death (hazard due to the cancer) were estimated using flexible Bayesian excess hazard models proposed by Quaresma *et al*.^21^ Separate models were fitted for men and women, adjusting for age at diagnosis, deprivation category and stage at diagnosis. To accommodate the hierarchical structure of the data (ie, that patients within a given CCG of residence or hospital of cancer care are likely to share some characteristics), the original model by Quaresma *et al* 21 was extended with the inclusion of a pair of random effects for CCG and hospital. To isolate the excess (cancer-related) hazards of death, the hazards of death from other causes were obtained for each patient with cancer from English life tables defined for each calendar year in 2006–2014 and stratified by single year of age, sex, deprivation category and region of residence.^22 23^ Five-year net survival for each CCG and hospital was estimated (based on the mean of their posterior distributions) and their variability across CCGs and hospitals was presented using funnel plots.^24^ Complete model specification details are presented in online supplemental appendix A. For this study, information on stage at diagnosis was not available for 23% of the cancer cases. In order to include all the cases in the analysis, we extended the excess hazard model to handle the missing stage information by specifying an additional distribution for the stage variable that uses information from all the covariates included in the main model specification. Additional analysis performed on complete cases confirmed the practical importance and the impact on results of accommodating the missing data structure in the analysis (see results in online supplemental appendix A.6, figure A.5). Conventional analyses were completed using Stata V.15,^19^ whereas Bayesian inferences were performed in R software V.3.4.3 using the JAGS MCMC program accessed via the R package ‘R2JAGS’.^25 26^

10.1136/jech-2021-217043.supp1 Supplementary data

## Results

**Figure 1:**
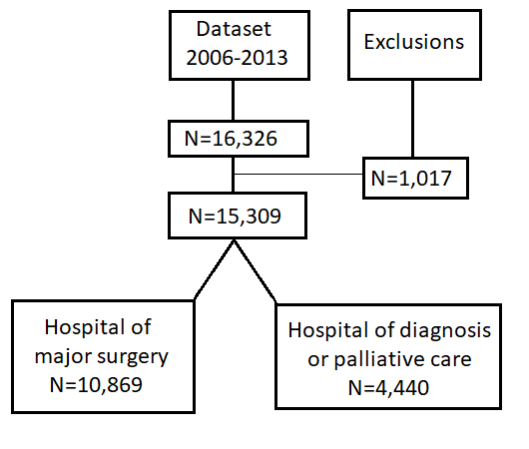
Flow chart of data exclusions and hospital assignment after applying the algorithm to allocate the hospital of care or diagnosis.

Data were available on 16 326 patients diagnosed with colon cancer between 2006 and 2013 in London, England (see flow chart in figure 1). For 15 309 (94%) patients, a hospital of cancer care was successfully allocated after the treatment capture algorithm was applied to each cancer record. The 1017 (6%) patients for which a hospital of cancer care or diagnosis could not be allocated were not included in further analyses. For 10 869 (71%) of the eligible 15 309 patients, the hospital allocated corresponded to the hospital where the patient underwent a major surgery for colon cancer. For the remaining 4440 (29%) patients, the hospital allocated corresponded either to the hospital of diagnosis provision or palliative care, if no major surgical treatment was recorded.

### Individual characteristics of patients with colon cancer by CCG and hospital

**Figure 2:**
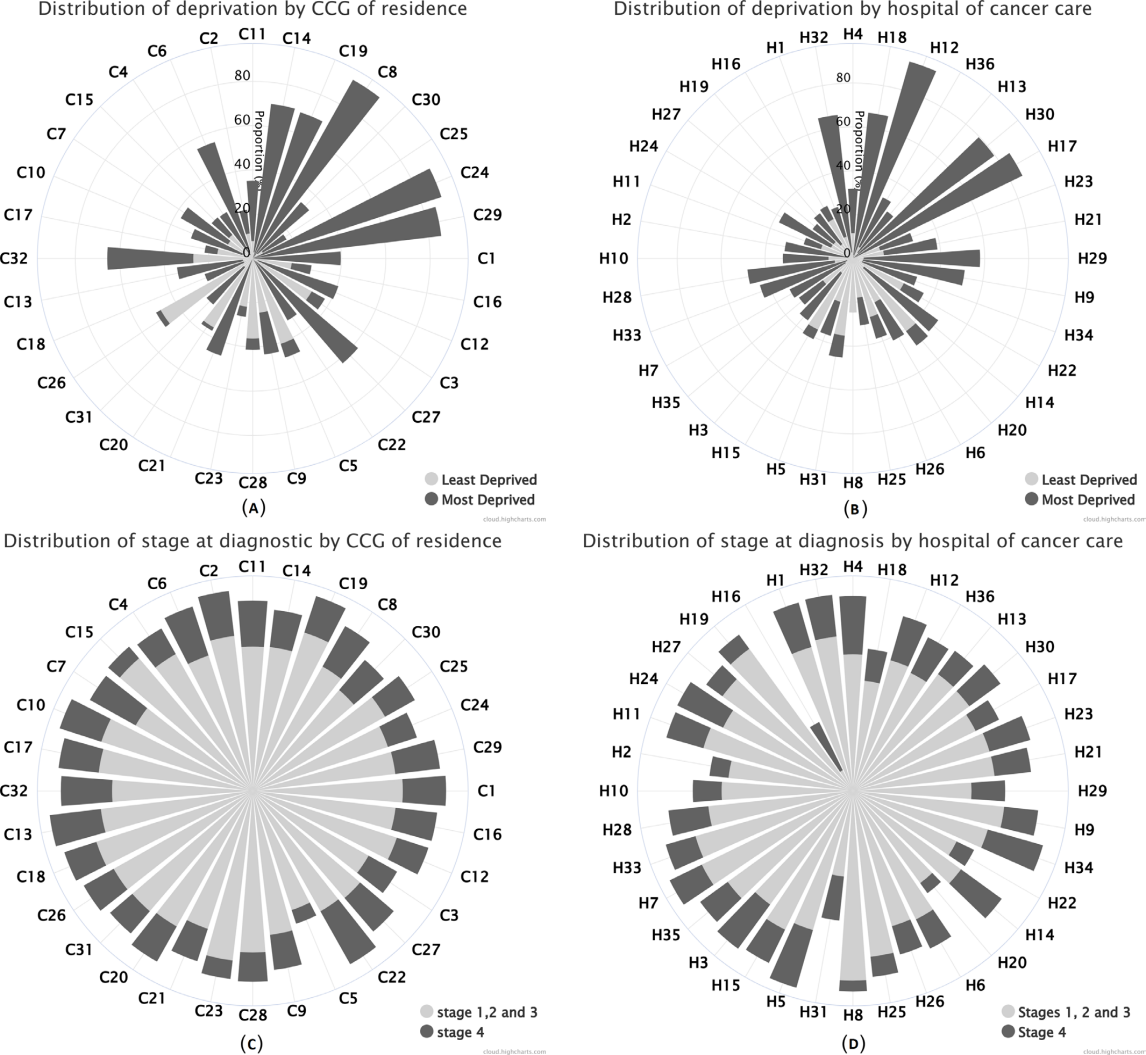
Windrose graphs showing the distribution (%) of male patients diagnosed with colon cancer in London, 2006–2013: (A) least deprived versus most deprived category by Clinical Commissioning Group (CCG) of residence; (B) least deprived versus most deprived category by hospital of cancer care; (C) stages at diagnosis 1, 2 and 3 versus stage 4 by CCG of residence; (D) stages at diagnosis 1, 2 and 3 versus stage 4 by hospital of cancer care.

Tables 1 and 2 show the distribution of cases and deaths for men and women by CCG of residence and NHS hospital of cancer care, respectively. Of the 15 309 patients included in the analysis, 7841 (51%) were men and 7468 (49%) were women. The number of cancer cases for patients living in a London CCG (and treated in a London hospital) within the study period ranged between 139 and 414 (median 227) for men and between 109 and 428 (median 217) for women. The number of cancer cases treated in an NHS hospital (and living in London) within the study period ranged between 30 and 435 (median 231) for men and between 18 and 469 (median 204) for women. Death was observed for 7674 (50%) patients over the maximum follow-up period of 8.9 years. Deaths ranged between 40% and 60% in both men and women for CCG of residence, and between 30% and 77% in men and between 38% and 75% in women for hospital of care. Survival time was measured from the date of diagnosis until the date of death or the date of last follow-up. For patients that died, the median survival time was 0.72 year, and for censored patients, the median survival time was 4.1 years. The mean age at diagnosis was 72 years (SD=13.2) for men and 74 years (SD=14.4) for women. For both men and women, the overall distribution of patients within deprivation categories was similar, ranging from 13% of patients in the least deprived group to 27% in the most deprived group. Stage at diagnosis was missing for 23% of the cases. Among the records with observed stage, the overall stage distribution was similar for both men and women, with 13% of patients diagnosed with stage 1 disease, 34% with stage 2, 34% with stage 3% and 19% of patients diagnosed with stage 4. The windrose graphs show that the highest proportion of patients from the most deprived group came from the North East/East London CCGs and hospitals, reaching over 80% of patients in some areas compared with the South West/South London areas where patients from the least deprived group are more predominant, although in much smaller proportions (figure 2A, B). The distribution of stages 1, 2 and 3 (grouped into one category) ranged between 50% and 73% by CCG of residence and between 37% and 80% by hospital of care. The distribution of patients with stage 4 was similar by CCG and hospital, ranging between 6% and 26% (figure 2C, D). These patterns were similar both for men and women.

**Table 1:** Number of cases (N) and proportion of deaths (%) within the follow-up period by CCG of residence for men and women diagnosed with colon cancer in London, 2006–2013

CCG of residence	Men		Women	
	Cases (N)	Deaths (%)	Cases (N)	Deaths (%)
C1: Barking and Dagenham	194	58.8	200	53.5
C2: Barnet	397	48.9	326	48.8
C3: Bexley	308	52.9	270	51.5
C4: Brent	263	44.9	240	49.2
C5: Bromley	414	54.1	428	53.3
C6: Camden	172	51.7	177	46.9
C7: Central London	156	54.5	109	41.3
C8: City and Hackney	187	49.7	176	51.1
C9: Croydon	401	46.9	369	50.7
C10: Ealing	308	50.0	303	47.2
C11: Enfield	308	51.3	323	49.8
C12: Greenwich	246	52.0	223	47.1
C13: Hammersmith and Fulham	154	48.7	160	46.2
C14: Haringey	212	50.9	204	52.9
C15: Harrow	234	42.7	221	44.8
C16: Havering	356	55.3	360	53.6
C17: Hillingdon	310	54.5	298	49.7
C18: Hounslow	215	41.9	206	50.0
C19: Islington	183	52.5	168	44.0
C20: Kingston	173	47.9	197	52.3
C21: Lambeth	240	44.2	245	47.3
C22: Lewisham	221	49.3	215	53.0
C23: Merton	218	48.6	217	50.2
C24: Newham	181	53.6	142	48.6
C25: Redbridge	268	50.4	275	50.9
C26: Richmond	252	43.6	225	46.7
C27: Southwark	218	50.9	214	54.2
C28: Sutton	254	45.7	253	49.0
C29: Tower Hamlets	139	59.7	142	54.9
C30: Waltham Forest	206	51.9	202	58.4
C31: Wandsworth	270	50.4	218	51.8
C32: West London	183	53.5	162	40.1
Total	7841	50.3	7468	50.0

CCG, Clinical Commissioning Group.

**Table 2:** Distribution of colon cancer cases and deaths for men and women, and EP by hospital of cancer care for patients diagnosed with colon cancer in London, 2006–2013

Hospital of cancer care	Men		Women		‘Internal’ patients		‘External’ patients	
	Cases (N)	Deaths (%)	Cases (N)	Deaths (%)	(%)	EP (%)	(%)	EP (%)
H1: Barnet Hospital	234	47.9	195	50.8	84.2	35.5	15.8	28.0
H2: Central Middlesex Hospital	48	68.7	48	75.0	76.0	56.7	24.0	69.2
H3: Charing Cross Hospital	169	49.7	177	43.5	39.0	36.9	61.0	24.7
H4: Chase Farm Hospital	182	59.3	193	49.2	88.8	35.6	11.2	25.7
H5: Chelsea and Westminster Hospital	180	51.7	180	42.2	40.0	29.0	60.0	33.7
H6: Croydon University Hospital	319	50.8	320	53.4	93.4	32.9	6.6	51.4
H7: Ealing Hospital	181	52.5	153	52.9	93.1	33.0	6.9	40.9
H8: Epsom Hospital	85	29.4	82	37.8	70.7	16.7	29.3	16.3
H9: Guy’s Hospital	88	37.5	90	42.2	32.0	20.8	68.0	25.0
H10: Hammersmith Hospital	72	65.3	68	55.9	30.0	37.1	70.0	27.8
H11: Hillingdon Hospital	245	57.9	247	51.4	95.7	37.0	4.3	61.1
H12: Homerton University Hospital	166	50.0	153	51.6	90.9	31.6	9.1	54.5
H13: King George Hospital	241	58.1	233	55.8	51.9	41.3	48.1	39.8
H14: King’s College Hospital	271	48.7	253	50.2	33.2	34.2	66.8	27.3
H15: Kingston Hospital	354	48.0	355	50.7	45.0	26.8	55.0	24.1
H16: Mount Vernon Hospital	30	76.7	18	61.1	72.9	0.0	27.1	0.0
H17: Newham General Hospital	142	57.7	128	50.8	92.2	39.6	7.8	45.0
H18: North Middlesex Hospital	187	55.6	176	57.4	49.6	34.7	50.4	38.1
H19: Northwick Park Hospital	236	42.4	209	46.9	31.7	39.7	68.3	36.6
H20: Princess Royal University Hospital	372	55.1	368	54.9	89.6	29.1	10.4	33.3
H21: Queen Elizabeth Hospital	339	52.2	277	49.1	58.4	35.3	41.6	34.1
H22: Queen Mary’s Hospital	178	57.9	187	55.6	74.5	24.6	25.5	29.5
H23: Queen’s Hospital	435	55.4	469	54.2	68.8	31.1	31.2	30.2
H24: Royal Free Hospital	235	51.5	217	49.8	33.9	39.4	66.1	27.1
H25: St. George’s Hospital	377	38.2	320	40.0	36.9	34.5	63.1	25.6
H26: St. Helier Hospital	234	58.9	244	59.8	61.9	48.5	38.1	40.8
H27: St. Mark’s Hospital	229	37.1	237	38.8	34.5	16.2	65.5	11.7
H28: St. Mary’s Hospital	265	39.2	227	38.3	25.2	30.2	74.8	22.6
H29: St. Thomas’ Hospital	247	55.1	230	53.9	32.5	40.3	67.5	29.7
H30: The Royal London Hospital	210	52.8	183	51.9	67.2	44.6	32.8	29.3
H31: The Royal Marsden Hospital	99	40.4	99	45.5	9.6	0.0	90.4	19.5
H32: The Whittington Hospital	181	56.3	199	47.2	47.4	40.0	52.6	40.2
H33: University College Hospital	283	40.9	244	39.3	27.9	26.6	72.1	19.9
H34: University Hospital Lewisham	181	47.5	180	49.4	78.4	26.7	21.6	18.8
H35: West Middlesex University Hospital	237	44.7	213	54.4	65.1	44.9	34.9	38.5
H36: Whipps Cross Hospital	309	50.8	296	53.4	61.5	30.1	38.5	21.9
Total	7841	50.3	7468	50.0	58.1	33.8	41.8	28.5

Proportion of patients with colon cancer and of EP according to which CCG the patients live in—within the CCG of each hospital (‘Internal patients’) or coming from other CCGs (‘External patients’).

CCGs, Clinical Commissioning Groups; EP, emergency presentation.

### Pathways of patients with colon cancer between their CCG of residence and hospital of cancer care

**Figure 3:**
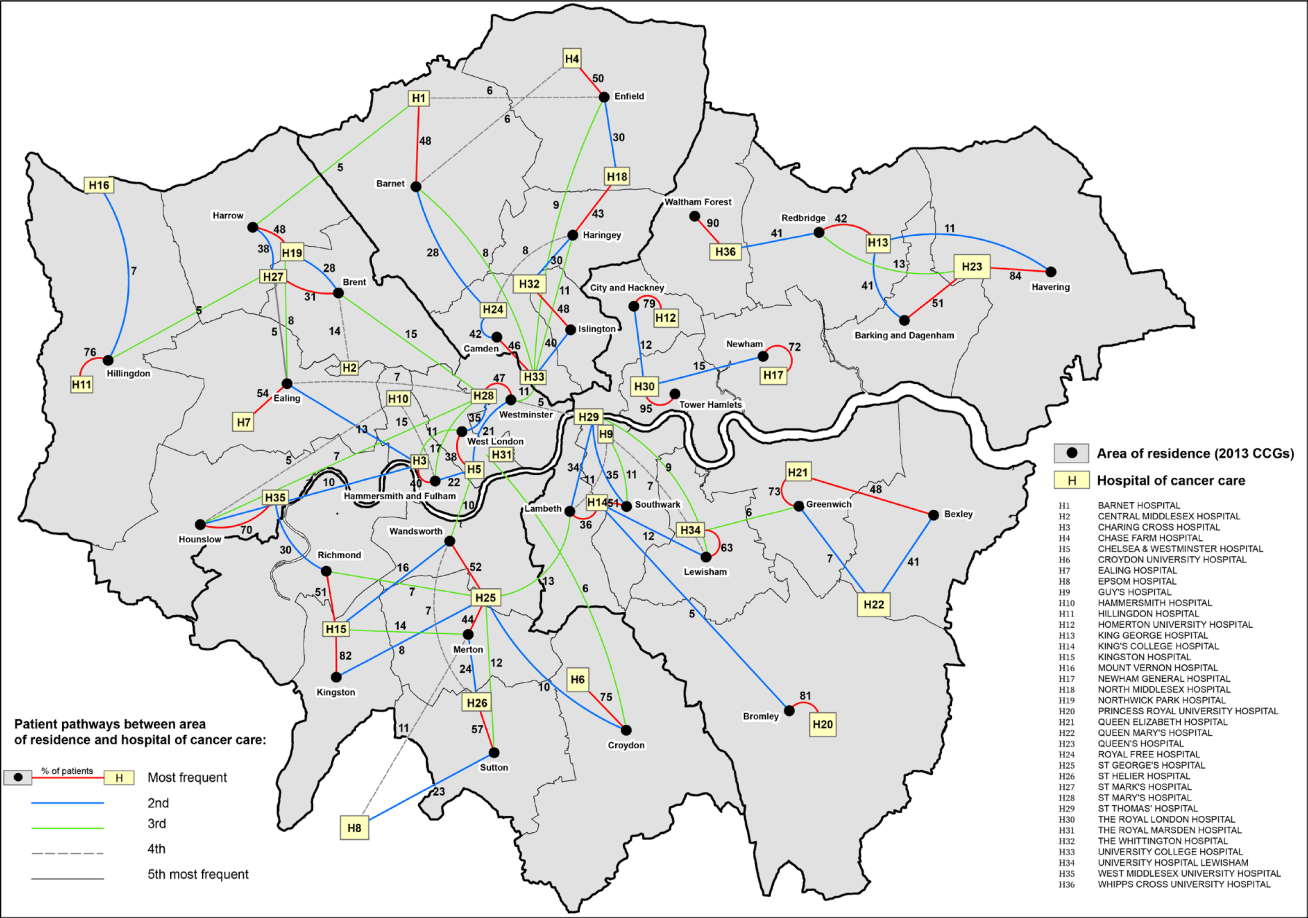
Flow map of London displaying the pathways of patients’ journeys between the area of residence (2013 Clinical Commissioning Groups (CCGs)) and the hospital of cancer care for men diagnosed with colon cancer, 2006–2013. Thick black borders define the boundaries for the CCGs as defined from 1 April 2021.

**Figure 4:**
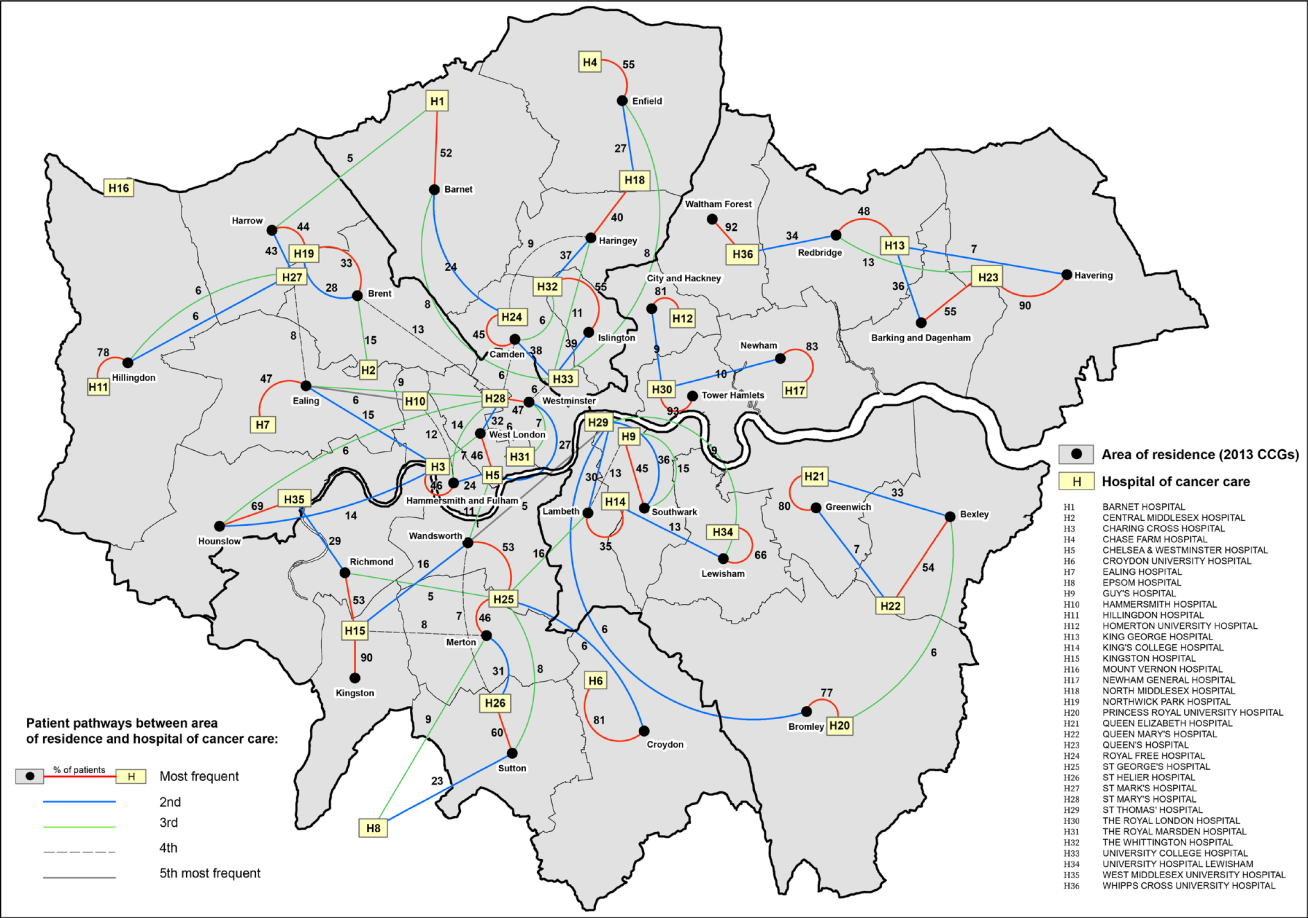
Flow map of London displaying the pathways of patients’ journeys between the area of residence (2013 Clinical Commissioning Groups (CCGs)) and the hospital of cancer care for women diagnosed with colon cancer, 2006–2013. Thick black borders define the boundaries for the CCGs as defined from 1 April 2021.

The flow maps in figures 3 and 4 display the pathways of patients between the CCG of residence and the hospital of cancer care for men and women, respectively. Overall, the most frequent pathway patients travelled was to the closest hospital located within the catchment area of their CCG of residence. Similar pathway frequencies were observed for both men and women. Three main patterns can be distinguished: (1) For one-third of CCGs, namely, Bromley, City and Hackney, Croydon, Greenwich, Havering, Hillingdon, Hounslow, Kingston, Newham, Waltham Forest and Tower Hamlets, more than 70% of patients travelled to one main hospital closest to their area of residence and with lower frequency to other hospitals. In particular, for patients living in Waltham Forest and Tower Hamlets (and Kingston for women), more than 90% travelled to only one hospital. (2) The second pattern identified CCGs in which patients travelled with similar frequency to two main hospitals close to their areas of residence, namely, Barking and Dagenham, Bexley, Camden and Islington. (3) For the remaining 17 CCGs, patients travelled more frequently up to three or four hospitals, travelling further to hospitals outside of their CCG of residence. Overall, the patterns displayed in the flow maps clearly define areas in London where patients’ travels are more self-contained to hospitals located in their neighbouring areas, for example, in the North East, East and South East of London. In contrast, patients living in the North and South West of London tend to access more hospitals outside their area of residence, most of them located in central London.

### Variations in 5-year cancer survival

**Figure 5:**
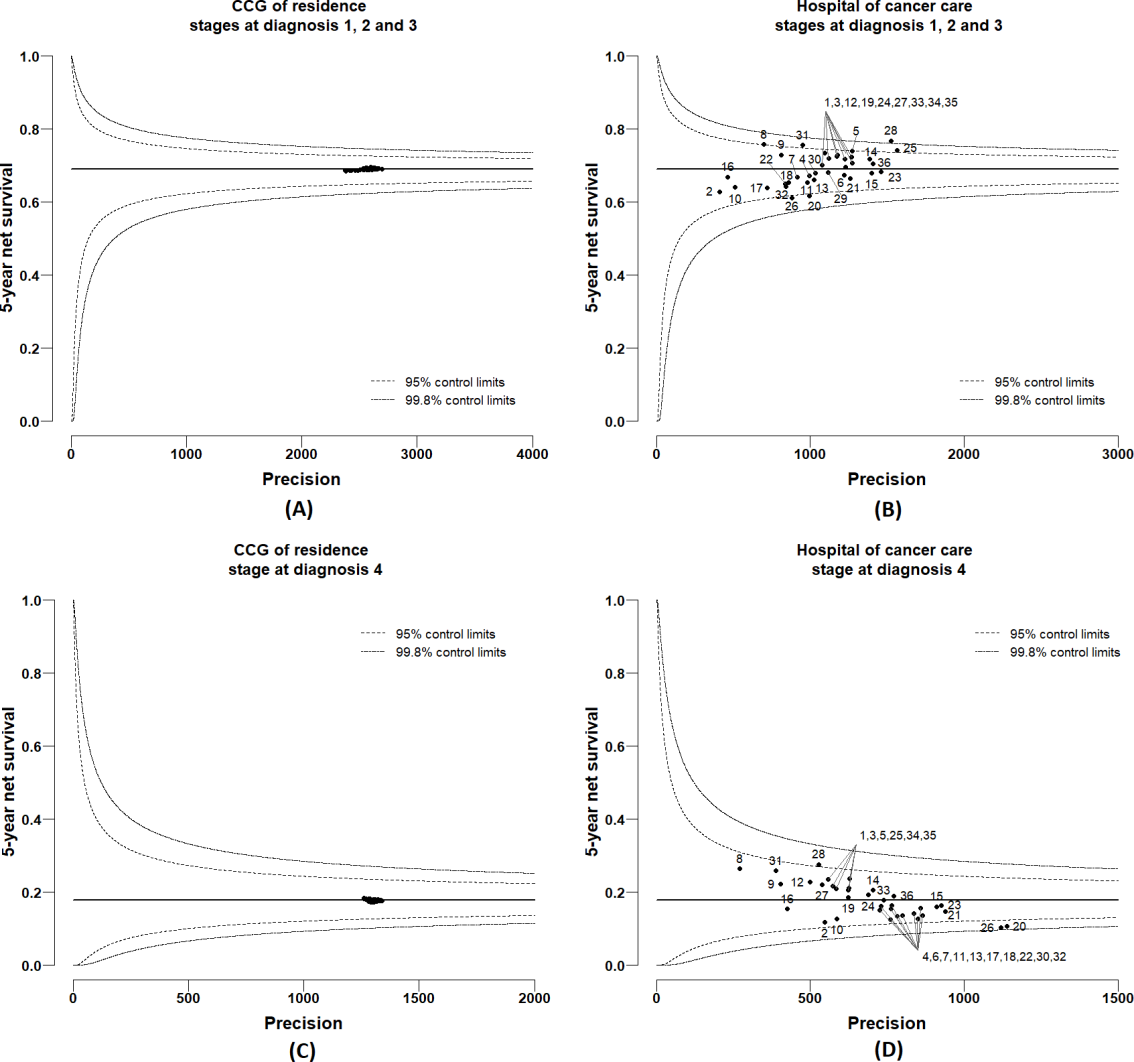
Funnel plots of 5-year net survival (mean posterior) for men diagnosed with colon cancer in 2006–2013, London: (A) by Clinical Commissioning Group (CCG) of residence for stages at diagnosis 1, 2 and 3; (B) by hospital of cancer care for stages at diagnosis 1, 2 and 3; (C) by CCG of residence for stage at diagnosis 4; (D) by hospital of cancer care for stage at diagnosis 4.

**Figure 6:**
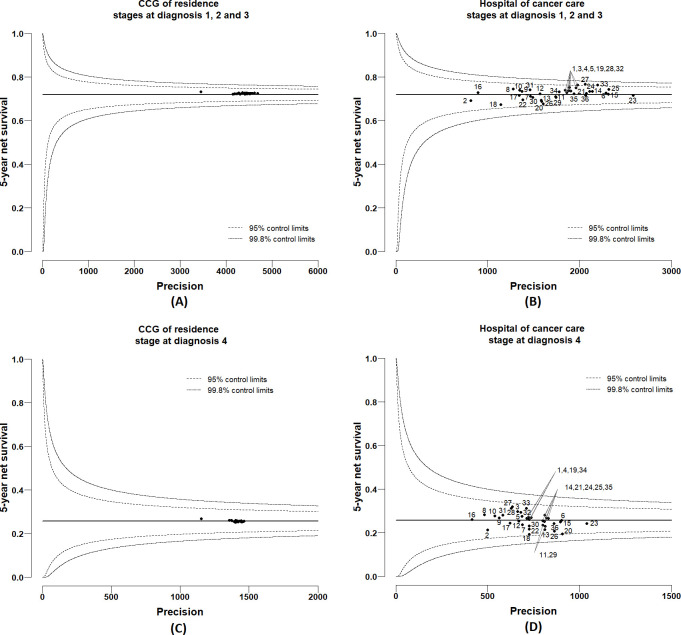
Funnel plots of 5-year net survival (mean posterior) for women diagnosed with colon cancer in 2006–2013, London: (A) by Clinical Commissioning Group (CCG) of residence for stages at diagnosis 1, 2 and 3; (B) by hospital of cancer care for stages at diagnosis 1, 2 and 3; (C) by CCG of residence for stage at diagnosis 4; (D) by hospital of cancer care for stage at diagnosis 4.

Posterior distributions of 5-year net survival were derived for each CCG of residence and hospital of cancer care from the multivariable excess hazard model, which included, in addition to CCG and hospital, age at diagnosis, deprivation and stage (full model). Complete model specification and Bayesian inference details are presented in online supplemental appendix A.1–A.5. From these posterior distributions, funnel plots were created by CCG of residence and hospital of care (figures 5 and 6) for men and women, respectively. Each funnel plot charts the 5-year net survival (posterior mean) against their corresponding precisions. Superimposed on the funnel plots are the 95% and 99.8% control limits. The target values (horizontal lines) were taken as the mean net survival for London. Net survival values that fall within the boundaries of the control limits are noted as consistent with the mean level of net survival in London. Net survival values that fall above the boundaries of the control limits exhibit more variability (with higher survival) than expected compared with the mean level of survival London, and values that fall below the boundaries of the control limits exhibit more variability (with lower survival) than expected compared with the mean level of survival in London. Plots were presented stratified by stage at diagnosis because the level of survival is very differential between early stages (stages 1, 2 and 3) and late stage (stage 4). No variability was observed between CCGs for both men and women (figures 5A, C and 6A, C), with all estimates almost exactly at the same level as the target line. However, large variability was observed between hospitals, although most of the estimates were contained within the 99.8% control limits in the funnel plots. For stages at diagnosis 1, 2 and 3, hospital-specific 5-year net survival ranged between 61% and 77% for men (with target 69%) (figure 5B) and between 67% and 76% for women (with target 72%) (figure 6B). For stage at diagnosis 4, the survival estimates ranged between 10% and 28% for men (with target 18%) (figure 5D) and between 19% and 32% for women (with target 26%) (figure 6D). For comparison of results with the full model, three additional excess hazard models were fitted by adding covariates successively: model 1, including age and CCG; model 2, including age, CCG and deprivation; model 3, including age, CCG, deprivation and stage. Based on each of these models, funnel plots were created by CCG of residence to visualise if any survival variability by CCGs was observed before the fully adjusted model. For both men and women, the 5-year net survival varied moderately between CCGs, even after adjusting for age at diagnosis, deprivation and stage at diagnosis (online supplemental figures 1a–c–4a–c). Such disparities disappeared once adjusted for hospital of cancer care, as shown by the funnel plots in online supplemental figures 1d–4d.

## Discussion

Our results reveal the complexity and multidimensionality of the association between the CCG-level colon cancer survival and hospital of cancer care for patients diagnosed with colon cancer during 2006–2013, who were living and receiving care in London, England. Flow maps of patient pathways between the CCG of residence and the hospital of cancer care revealed that patients travelled more frequently to hospitals closest to their area of residence, in particular in the North East, East and South East of London, whereas patients living in the North and South West of London frequently accessed hospitals outside their area of residence. The differential frequencies in patient pathways between area of residence and hospital of care raise questions regarding the equal choice of patients for the best performing hospitals at point of referral. Moderate variation in the 5-year net survival was observed between CCGs after adjusting for age at diagnosis, deprivation and stage at diagnosis. These disparities disappeared once adjusted for hospital of cancer care, while substantial variation between hospitals emerged after adjusting for the same patient-level and tumour-level factors. Overall, we observed a strong correlation between the net survival values estimated in the CCGs and their local hospitals. It is nevertheless crucial for interpretation purposes to highlight the weak correlation between some CCGs and their local hospitals. In particular, some of the hospitals located in CCGs with poorer net survival do not seem to be associated with lower survival. Further examination of the patient flow and the levels of net survival revealed a few points with potentially important policy implications. Bromley, Newham, Tower Hamlets and Waltham Forest combine (1) some of the lowest CCG level 5-year net survival estimates (below 68%) for patients with colon cancer residing in these London CCGs, and (2) some of the highest proportions of patients treated within the CCG (above 70% and even above 90% for Tower Hamlets and Waltham Forest) (figures 3 and 4). Hospital-level outcomes, however, differ notably: survival of patients with colon cancer managed by the local hospitals of Tower Hamlets and Waltham Forest compared well with the London average, whereas local hospitals of Bromley and Newham presented some of the poorest levels of net survival in London (figures 5 and 6). The hospitals of Tower Hamlets and Waltham Forest manage large proportions of patients with colon cancer coming from other CCGs and those ‘external patients’ had differential characteristics from those who are managed in these hospitals and live within these CCGs (‘internal patients’) (table 2). Among the external patients, the proportions of EP were around 29% and 22% in each local hospital, while the figures were 45% and 30% for the internal patients, respectively. These findings suggest that the care provided by these hospitals to elective patients is as expected, but they also raise questions regarding the effective implementation of cancer policies for the populations within these CCGs, given that high EP proportions commonly reflect longer referral times, poor access to specialists, difficulties in accessing diagnostic tests and barriers in communication between primary and secondary care.^27^ In addition, the deprivation level is high in most of the populations living in these CCGs (ranging between 30% and 85% of the population in the most deprived group), meaning that these patients, on average, have low awareness for cancer symptoms,^28^ experience barriers to help seeking^29^ and have more comorbidities, which complicates both the diagnosis^30^ and the treatment of the cancer. In particular, one may wonder whether the shortness of primary care consultations does not penalise patients embarrassed or who struggle to describe their symptoms and communicate their choices. The implementation of the 2-week referral pathway does not seem very successful in these areas. The situation in Bromley and Newham CCGs strongly contrasts with the previous CCGs as their local hospitals presented some of the poorest levels of net survival in London with high levels of EP (30% and 40%, respectively). This suggests that in addition to the previously mentioned EP challenges that these populations face, the performance of their local hospitals and cancer outcomes are very poor even for elective patients, raising questions regarding the internal organisation and availability of resources these hospitals have for an effective cancer management.

To the best of our knowledge, this is the first study to investigate in-depth variation in cancer survival at both area of residence and hospital level. The timing of our findings remains relevant in the light of both the current COVID-19 pandemic and the recent successive regroupings of CCGs. The pandemic has led to the temporary suspension of cancer screening services and deferred routine diagnostic work, only prioritising urgent symptomatic cases for diagnostic intervention. We expect that these diagnostic delays will almost surely continue to exacerbate the disparities we have observed in our study for the foreseeable future. A recent study suggests that as a result of these unprecedented diagnostic delays, substantial increases in the number of avoidable cancer deaths in England are to be expected, and stress the need for urgent policy interventions to mitigate the expected impact of the COVID-19 pandemic on patients with cancer.^31^ Our results have shown how the access to cancer care can be challenging for populations living in some specific areas and it is crucial to monitor how this will be modified by the reorganisation of London CCGs. A close monitoring is even more necessary as the new CCG grouping seems to strongly reinforce the geographical disparities regarding the sociodemographic and the cancer outcomes (see figures 3 and 4). The large size of the new CCGs poses the question of how geographical inequalities should be monitored and investigated. Furthermore, we advocate caution when interpreting the hospital-specific net survival estimates presented in this study, as these hospitals treat more patients than the selected cohort of patients with cancer here analysed.

In summary, this study demonstrates the importance of performing more in-depth investigations into the observed disparities in cancer survival using population-based data enriched with other relevant health data sources. The results presented here pertain only to a small part of England, but they reveal a very complex picture of large variation across London between access of patients with cancer to specialist care and effective delivery of such care. We hypothesise that similar patterns exist in the rest of the country, and future research should aim to expand a similar analysis to the whole of England as a means to inform national policy makers. Such an analytical approach would also be very informative in other countries with comparable settings. Future work should also aim to investigate hospitals with poorer performance to understand its causes (including their resources and organisation), and to examine more in depth (including qualitative studies) what determines the choice (or absence of choice) of patients for a given hospital in order to suggest actions to correct such wide disparities.

**What is already known on this subject**

- Wide geographical inequalities in survival from most cancer types have been consistently described in England, with similar disparities observed within the capital London, despite the existence of free-of-charge care within the National Health Service.

**What this study adds**

- Access to cancer care revealed itself challenging for populations living in specific areas of London, and our results highlight the need to better coordinate primary and secondary care sectors in order to improve timely access to specialised clinicians and diagnostic tests.
- Close monitoring of cancer outcomes remains crucial in the light of the most recent reorganisation of Clinical Commissioning Groups and the observed exacerbation of disparities during the COVID-19 pandemic, not only in London but also at national level and for other cancer types.

## Data Availability

Data may be obtained from a third party and are not publicly available. This study used English cancer registry data. The authors do not own these data and hence are not permitted to share them in the original form. The data are available from the Office for Data Release at Public Health England. For access, please email odr@phe.gov.uk.
