# Supplementary material for: Variation in colon cancer survival for patients living and receiving care in London, 2006–2013: does where you live matter?
Source: J Epidemiol Community Health. 2021 Aug 16;76(2):196–205. doi: 10.1136/jech-2021-217043 (PMC8762004; doi:10.1136/jech-2021-217043)
Supplement: Supplementary data [file jech-2021-217043supp001.pdf]

Appendix A. Flexible Bayesian hierarchical excess hazard models

Appendix A.1. Model specification

Excess hazard models were set-up for men and women, including age at diagnosis (AGE), deprivation category (DEP), stage at diagnosis (STAGE), CCG of residence (CCG) and hospital of care (HOSP). The models were defined on the log-excess hazard scale ($log(h_E(t))$) and use low-rank thin plate (LRTP) splines to model the smooth effect of the baseline excess hazard and the smooth effect of age at diagnosis.[1] The observed follow-up time (t) was divided into four partitions (K=4), chosen at the 25%, 50% and 75% percentiles of the event (death) times. For men these were chosen at $\widetilde{t}$=(0, 0.28, 1.08, 2.4, 8) years and for women at $\widetilde{t}$=(0, 0.27, 1, 2.3, 8) years. Both models, for men and women, were formulated as

$$\begin{aligned} log(h_E(t|\alpha;\beta;\gamma;\nu;\iota;\zeta)) = {} & (\alpha_{0,0} + \alpha_{1,0}AGE) + (\alpha_{0,1} + \alpha_{1,1}AGE)t \\ & + \sum_{k=2}^{K}(\alpha_{0,k} + \alpha_{1,k}AGE)(|t - \tilde{t}_{k-1}| - |\tilde{t}_{k-1}|) \qquad \text{[part 1]} \\ & + \beta_1^*(AGE - \overline{AGE}) + \sum_{j=2}^{J}\beta_j^*(|AGE - \widetilde{AGE}_{j-1}|^3 \\ & - |\overline{AGE} - \widetilde{AGE}_{j-1}|^3) \qquad \text{[part 2]} \\ & + \sum_{l=2}^{5}(\gamma_l * DEP_l) \qquad \text{[part 3]} \\ & + \nu * STAGE \qquad \text{[part 4]} \\ & + \sum_{v=1}^{32}(\iota_v * CCG_v) \qquad \text{[part 5]} \\ & + \sum_{h=1}^{36}(\zeta_h * HOSP_h) \qquad \text{[part 6]} \end{aligned} \tag{A.1}$$

where, [part 1] formulates the LRTP spline modelling the baseline log-excess hazard, incorporating the time-dependent effect of age at diagnosis using the same follow-up time partition, with parameters $\alpha = (\alpha_0|\alpha_1)$ and $\alpha_q = (\alpha_{q,0}, \ldots, \alpha_{q,K})$ for q=0,1. [part 2] represents the LRTP spline modelling the

non-linear (smooth) effect of age at diagnosis using 3 partitions (J=3) of the observed age range at $\widetilde{AGE}$=(15, 43, 71, 99) years, for both men and women, with parameters β_j, $j = 1, \ldots, J$. $\overline{AGE}$ represents the mean age at diagnosis. For ease of interpretation, age at diagnosis was centered at age 70. [part 3] formulates the effect of deprivation modelled as a categorical variable (DEP_1: least deprived to DEP_5: most deprived), with parameters γ_l, $l = 2, \ldots, 5$. The least deprived group (DEP_1) was set as the baseline fixing $\gamma_1 = 0$. [part 4] formulates the effect of stage at diagnosis modelled as a binary variable (STAGE=0 for stages 1, 2 and 3 grouped and STAGE=1 for stage 4), with parameter ν. [part 5] defines the random effects for CCG of residence, with parameters ι_v, $v = 1, \ldots, 32$. [part 6] defines the random effects for hospital of care, with parameters ζ_h, $h = 1, \ldots, 36$.

Appendix A.2. Prior distributions

Prior distributions for the model parameters were chosen as:

- For the baseline log-excess hazard, including the time dependent effect of age at diagnosis ([part1]):

$$\begin{gathered}\alpha_{q,0} \sim N(0, 10^4)\ ,\ \alpha_{q,1} \sim N(0, 10^4) \text{ for q=0, 1} \\ \alpha_{q,k}|\sigma_{q,\alpha} \overset{iid}{\sim} N(0, \sigma^2_{q,\alpha}) \text{ for k=2, } \ldots \text{ , K and } \sigma_{q,\alpha} \sim U(0.01, 100) \text{ for q=0, 1}\end{gathered} \tag{A.2}$$

- For the non-linear effect of age at diagnosis ([part 2]):

$$\begin{gathered}\beta_0 \sim N(0, 10^4) \\ \beta_k|\sigma_\beta \overset{iid}{\sim} N(0, \sigma^2_\beta)\ \text{, for k=2, } \ldots \text{ , K and } \sigma_\beta \sim U(0.01, 100)\end{gathered} \tag{A.3}$$

- For the effect of deprivation ([part 3]):

$$\begin{gathered}\gamma_0 = 0 \\ \gamma_l|\sigma_\gamma \overset{iid}{\sim} N(0, \sigma^2_\gamma)\ \text{, for l=2, } \ldots \text{ , 5 and } \sigma_\gamma \sim U(0.01, 100)\end{gathered} \tag{A.4}$$

- For the effect of stage at diagnosis ([part 4]):

$$\nu \sim N(0, 10^4) \tag{A.5}$$

- For the random effects on CCG of residence ([part 5]):

$$\iota_v|\sigma_\iota \overset{iid}{\sim} N(0,\sigma_\iota^2) \text{ , for v=1, } \dots \text{ , 32 and } \sigma_\iota \sim U(0,10) \tag{A.6}$$

- For the random effects on hospital of cancer care ([part 6]):

$$\zeta_h|\sigma_\zeta \overset{iid}{\sim} N(0,\sigma_\zeta^2) \text{ , for h=1, } \dots \text{ , 36 and } \sigma_\zeta \sim U(0,10) \tag{A.7}$$

Appendix A.3. Handling missing information on stage at diagnosis

Information on stage at diagnosis was missing for 22% of men and 24% of women in the dataset analysed in this study. All other variables had no missing information. In order to include all the cases in the analysis, we extended the model specified in Appendix A.1 to define a prior distribution for stage at diagnosis using a Bernoulli distribution with probability μ as

$$STAGE \sim Bernoulli(\mu) \tag{A.8}$$

and we defined as a prior distribution for μ a logistic regression model including all the covariates used in the main model to better impute the missing stage information as

$$\begin{aligned} logit(\mu) = \lambda_1 * AGEi + \sum_{l=2}^{5}(\lambda_{2l} * DEP_l) \\ + \sum_{v=1}^{32}(\lambda_{3v} * CCG_v) + \sum_{h=1}^{36}(\lambda_{4h} * HOSP_h) \end{aligned} \tag{A.9}$$

where, $AGEi$ is now modelled as a linear effect of age at diagnosis, with parameter λ_1. The effects of deprivation with parameters λ_{2l}, of CCGs with parameters λ_{3v} and of hospitals with parameters λ_{4h} are modelled in the same way as in the main model formulation (Appendix A.1). Prior distributions for all the λ parameters were defined as

$$\begin{aligned} \lambda_1 &\sim N(0,0.0001) \\ \lambda_{2l} &\overset{iid}{\sim} N(0,0.0001) \text{ , for l=2, } \dots \text{ , 5} \\ \lambda_{3v} &\overset{iid}{\sim} N(0,0.0001) \text{ , for v=1, } \dots \text{ , 32} \\ \lambda_{4h} &\overset{iid}{\sim} N(0,0.0001) \text{ , for h=1, } \dots \text{ , 36} \end{aligned} \tag{A.10}$$

Appendix A.4. Bayesian inference

Bayesian inferences were performed in R software version 3.4.3 using the JAGS MCMC program accessed via the R package 'R2JAGS'.[2, 3] Models were fitted setting up 2 MCMC chains, each with 60,000 iterations, a burn-in period of 10,000 and a thinning of 2 to eliminate any potential autocorrelation among samples within the chains. A total of 50,000 sampled values were retained from the posterior distributions of each of the model parameters. An examination of the trace and density plots of each parameter's posterior distribution did not indicate any convergence issues for these samples. The 50,000 sampled values from the parameter posterior distributions were used to derive posterior distributions of 5-year net survival for each CCG of residence and hospital of care. These were derived using a 'prediction matrix' that included all the combinations of age at diagnosis (individual integer ages within the observed age range 15-99 years), deprivation category (1-5), stage at diagnosis (0-1), CCG (32) and hospital (36).

Appendix A.5. Funnel plots for the additional models fitted

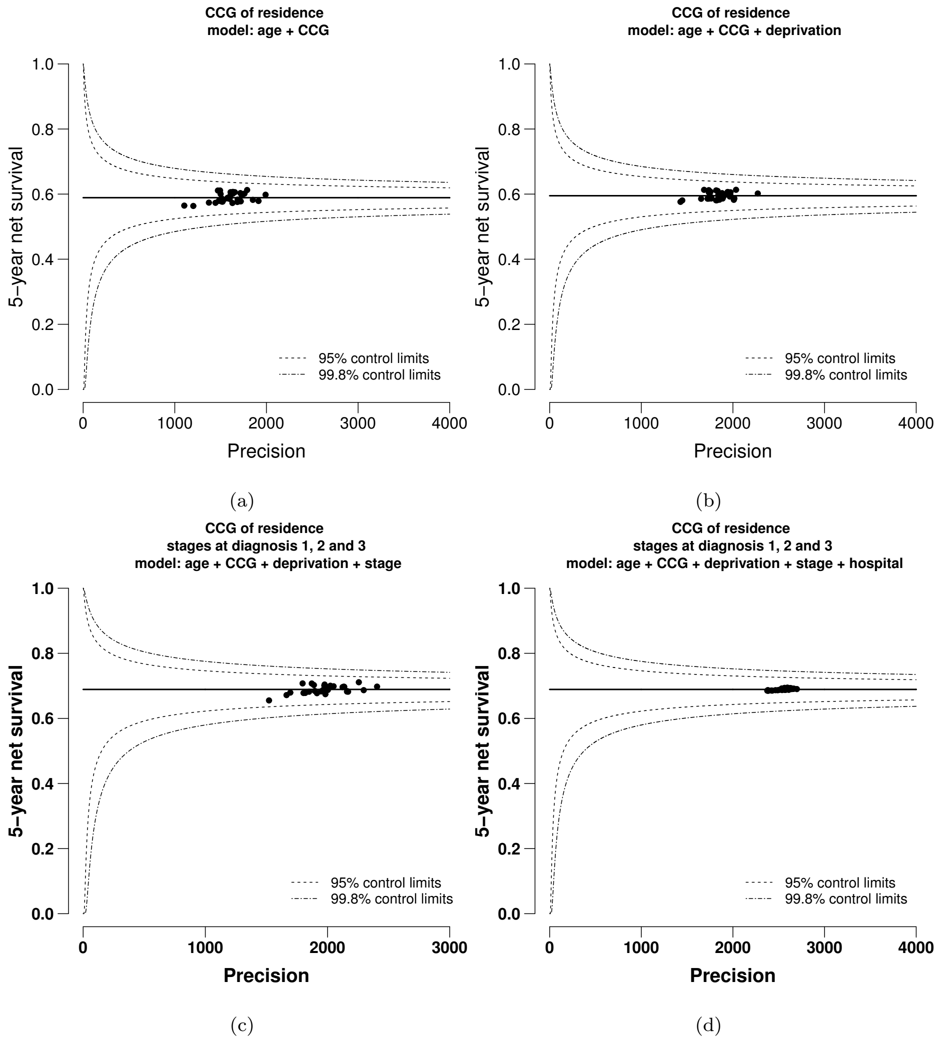

Figure A.1: Funnel plots of 5-year net survival by CCG of residence (mean posterior) for men diagnosed with colon cancer in 2006-2013, London: (a) model including age at diagnosis and CCG (b) model including age at diagnosis, CCG and deprivation; (c) model including age at diagnosis, CCG, deprivation and stage at diagnosis (for stages at diagnosis 1, 2 and 3); (d) model including age at diagnosis, CCG, deprivation, stage at diagnosis and hospital of cancer care (for stages at diagnosis 1, 2 and 3).

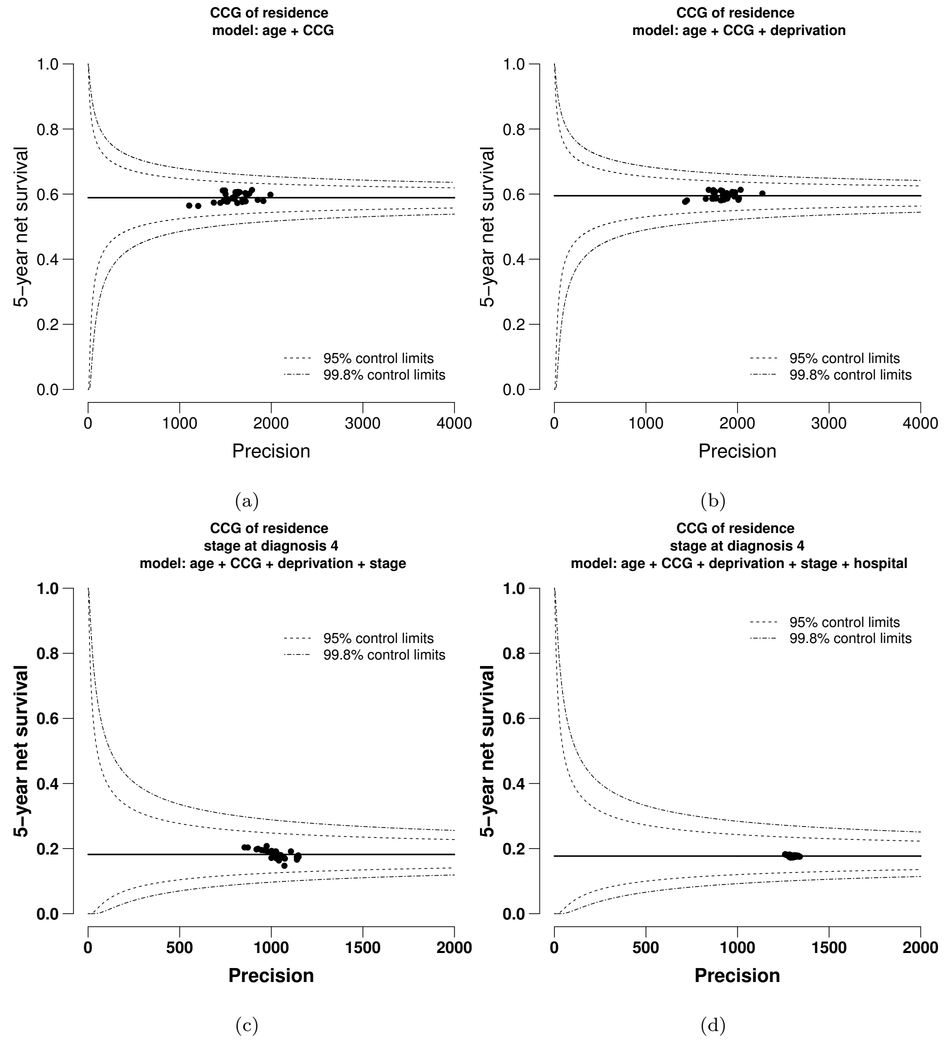

Figure A.2: Funnel plots of 5-year net survival by CCG of residence (mean posterior) for men diagnosed with colon cancer in 2006-2013, London: (a) model including age at diagnosis and CCG (b) model including age at diagnosis, CCG and deprivation; (c) model including age at diagnosis, CCG, deprivation and stage at diagnosis (for stage at diagnosis 4); (d) model including age at diagnosis, CCG, deprivation, stage at diagnosis and hospital of cancer care (for stage at diagnosis 4).

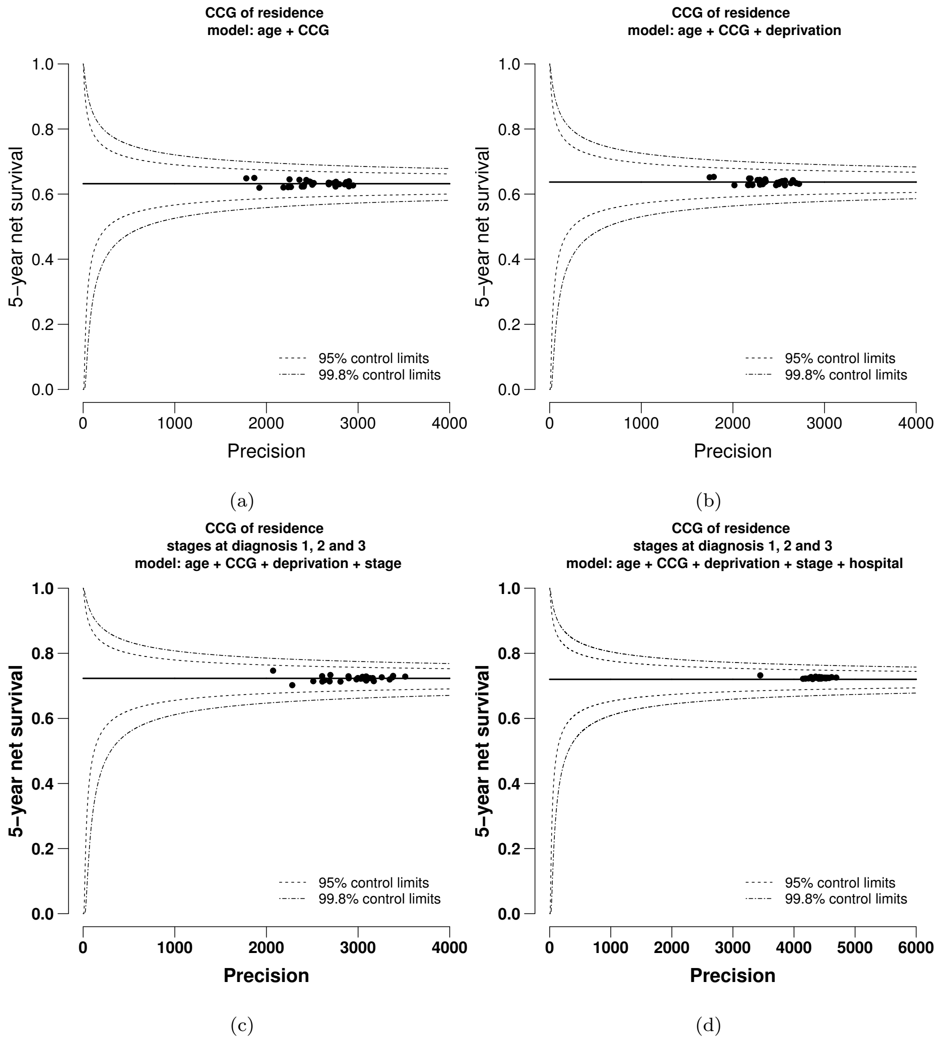

Figure A.3: Funnel plots of 5-year net survival by CCG of residence (mean posterior) for women diagnosed with colon cancer in 2006-2013, London: (a) model including age at diagnosis and CCG (b) model including age at diagnosis, CCG and deprivation; (c) model including age at diagnosis, CCG, deprivation and stage at diagnosis (for stages at diagnosis 1, 2 and 3); (d) model including age at diagnosis, CCG, deprivation, stage at diagnosis and hospital of cancer care (for stages at diagnosis 1, 2 and 3).

Quaresma M, *et al. J Epidemiol Community Health* 2021; 76:196–205. doi: 10.1136/jech-2021-217043

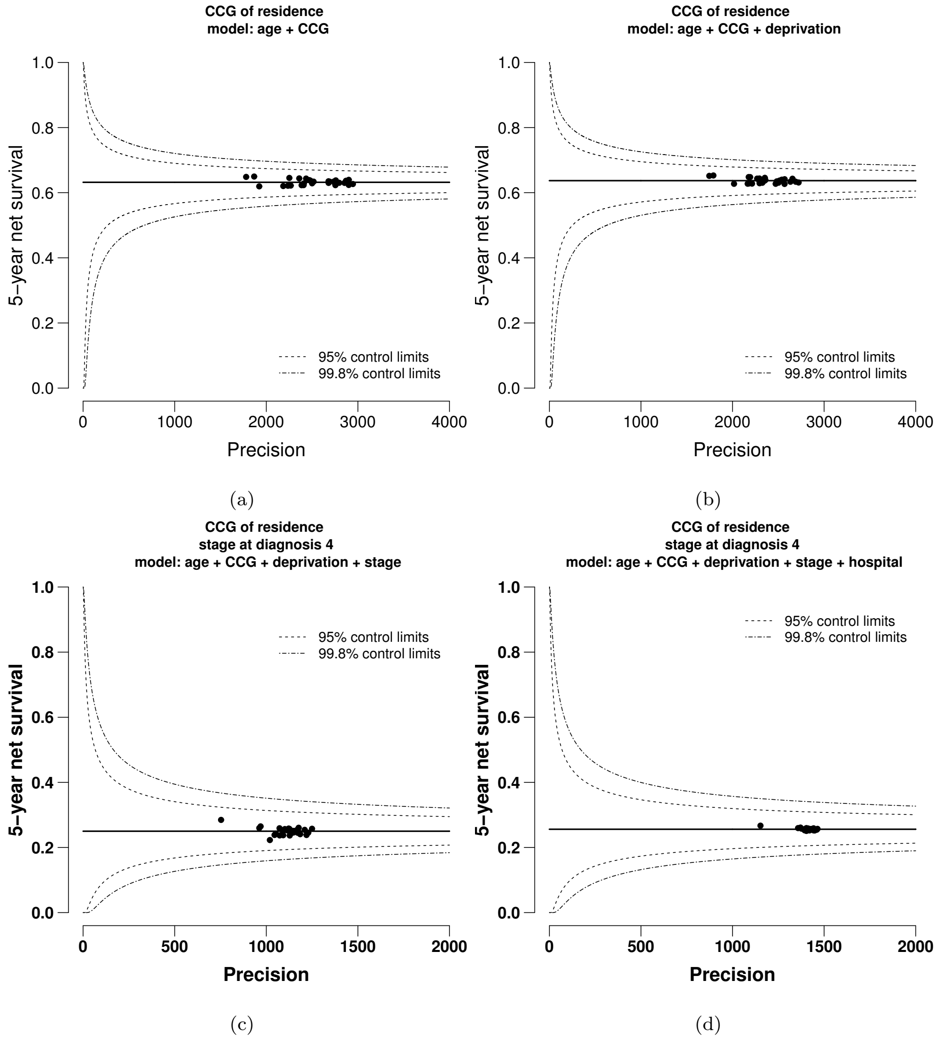

Figure A.4: Funnel plots of 5-year net survival by CCG of residence (mean posterior) for women diagnosed with colon cancer in 2006-2013, London: (a) model including age at diagnosis and CCG (b) model including age at diagnosis, CCG and deprivation; (c) model including age at diagnosis, CCG, deprivation and stage at diagnosis (for stage at diagnosis 4); (d) model including age at diagnosis, CCG, deprivation, stage at diagnosis and hospital of cancer care (for stage at diagnosis 4).

Quaresma M, *et al. J Epidemiol Community Health* 2021; 76:196–205. doi: 10.1136/jech-2021-217043

Appendix A.6. Funnel plots for the complete case analysis versus modelling missing data

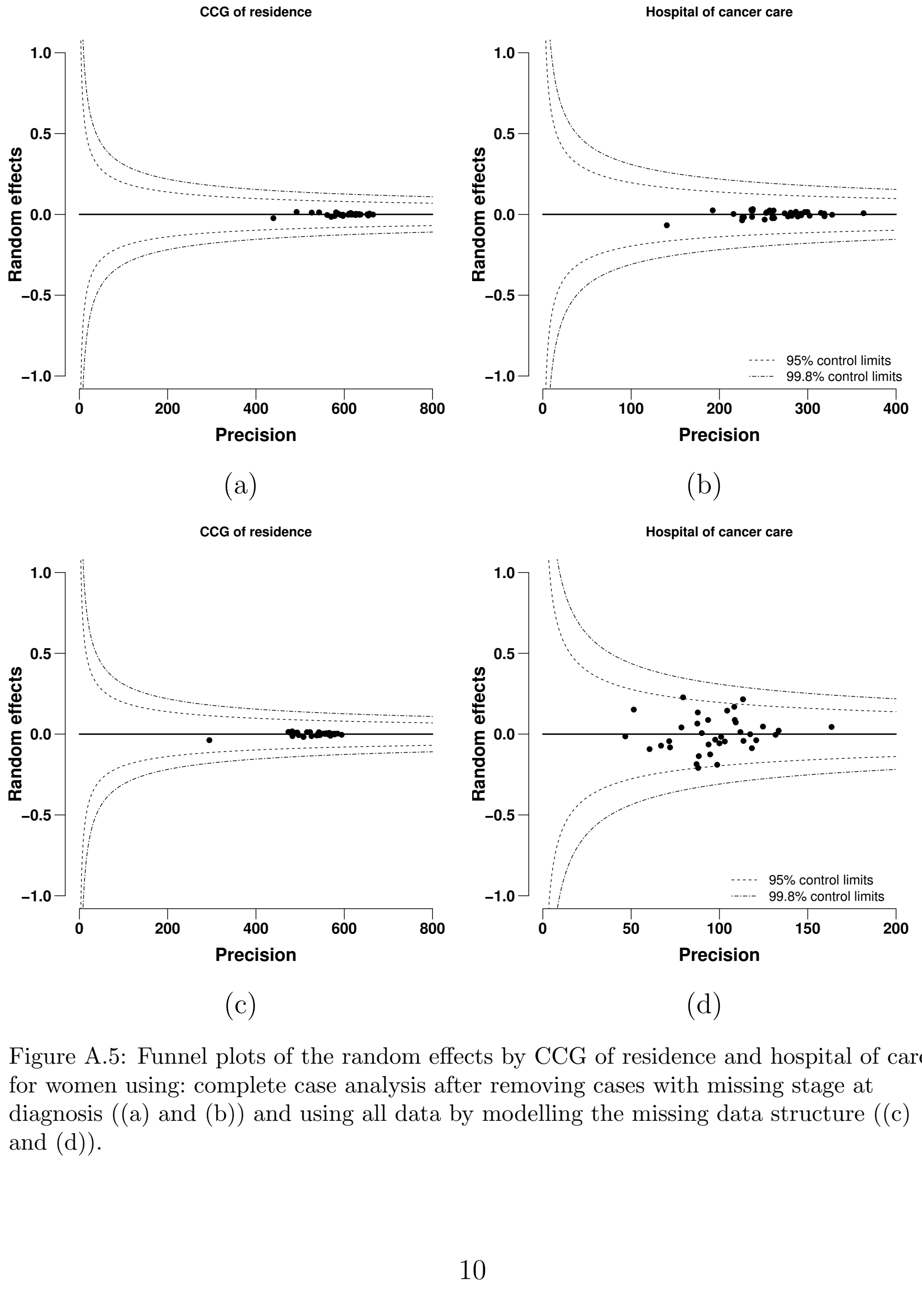

Figure A.5: Funnel plots of the random effects by CCG of residence and hospital of care for women using: complete case analysis after removing cases with missing stage at diagnosis ((a) and (b)) and using all data by modelling the missing data structure ((c) and (d)).

References

[1] Quaresma, M., Carpenter, J., Rachet, B. 2019. Flexible bayesian excess hazard models using low-rank thin plate splines. Statistical Methods in Medical Research. 2020;29(6):1700-1714. doi: `https://doi.org/10.1177/0962280219874094`.

[2] RStudio Team. RStudio: Integrated Development for R. 2017. url: `http://www.rstudio.com/`.

[3] Plummer, M. JAGS: A program for analysis of Bayesian graphical models using Gibbs sampling. 2003. url: `http://www.ci.tuwien.ac.at/Conferences/DSC-2003/Drafts/Plummer.pdf`.
